# Monthly Summary

**Published:** 1899-09

**Authors:** 


### Monthly Summary.

ALVEOLAR HEMORRHAGE.—Having read the articles on "Alveolar Hemorrhage" in the May and June "Items of Interest" I wish to report a case that came under my observation. My case was similar to Dr. Benjamin's, reported in the May number.

In the fall of 1887, I extracted the upper teeth for a maiden lady preparatory to a plate. There was nothing unusual in the force used. The extraction was done in the forenoon. In the afternoon, about three o'clock they reported to me that the lady was suffering from severe hemorrhage. As she lived only across tbe street from my office, I went with them to her home. Thinking that the bleeding was from a few sockets only, I packed them with cotton and tannic acid. The hemorrhage did not subside. The family were very much alarmed, and asked me if they could send for their family physician. I replied, certainly, but that I should like to be called when he arrived.

In about an hour's time Dr. C. came, and I was noticed. Dr. C. was using medicine, and the mouth was so full of clotted blood that the lady could hardly swallow. An attempt of thirty or forty minutes failed to arrest the hemorrhsge. The physician then turned the patient over to me. Having thoroughly cleansed the mouth, I took a wax impression of the upper jaw. I made a plaster cast formed a trial plate of gutta percha. Then a roll of gutta percha, quite soft, was placed on the trial plate directly over the sockets. I placed the trial plate with the softened gutta percha rim in her mouth, and had her bite into it. After hardening the gutta-percha with cold water I bendaged the jaws. Within a few minutes the bleeding ceased, and did not recur when my compress was removed the next day.

In twenty years' practice this is the only case of hemorrhage I have had where all the teeth were extracted.

My only claim for originality is in the use of a compress for the whole upper jaw.—*Items of Interest.*

---

TO APPLY THE RUBBER DAM.—In order to get the rubber-dam well up on the neck of the tooth, so as to obtain a clear view of the cervical margin of the cavity about to be filled, wrap a piece of dental floss or fiilling thread twice around the tooth, and push this well up on the neck of the tooth, then tie. Let this remain for a couple of days, and when the patient returns, the application of the dam can be made with ease to the operator and with little discomfort to the patient, affording a perfect view of the parts to be operated on. Should there be a large space between the teeth, the interdental space *below the ligature* may be filled with cotton steeped in sandarac varnish, or with pink gutta-percha.—*Dental Office and Laboratory.*

---

ARSENICAL POISONING CURED BY ORTHOFORM.—I had a case of acute arsenic poisoning with the conditions present so familiar to all. With spoon excavator removed the blackened gum tissue, syringed parts with warm antiseptic solution, soaked gum and surroundings with dialysed iron, and applied twenty-five per cent. unguent orthoform. The pain in this case was the severest I ever saw, and I must admit being astonished when the patient returned on the following day and reported that pain stopped and not returned. Relief came within twenty minutes.—*A. D. Kyner, Items.*

